# Background and Clinical Features of a Unique and Mysterious Autoinflammatory Disease, Schnitzler Syndrome

**DOI:** 10.3390/ijms26020598

**Published:** 2025-01-12

**Authors:** Györgyi Műzes, Ferenc Sipos

**Affiliations:** Immunology Division, Department of Internal Medicine and Hematology, Semmelweis University, 1088 Budapest, Hungary

**Keywords:** Schnitzler syndrome, autoinflammatory disease, urticaria, monoclonal IgM, canakinumab, anakinra, diagnosis, MEFV gene, F2 gene

## Abstract

Schnitzler syndrome is a unique autoinflammatory disease, of which 747 cases have been described worldwide to date. The main features of the syndrome are a triad of recurrent urticaria, monoclonal IgM gammopathy, systemic inflammation associated with recurrent fever, joint and bone pain, and atypical bone remodeling (osteosclerosis). The abnormal activation of the NLRP3 inflammasome produces IL-1, which drives the disease pathology, but it also involves IL-6 and IL-18. Unlike other autoinflammatory diseases, Schnitzler syndrome lacks evidence of the gene divergence causing the abnormal activation of NLRP3. However, mutations in the MEFV and MYD88 genes can be associated with the development of the disease. Due to its rarity, diagnosing the disease can be a challenging task. IL-1 inhibitors (i.e., anakinra, canakinumab, and rilonacept) are prominent in the treatment of the disease, but the IL-6 receptor inhibitor tocilizumab and the Bruton’s tyrosine kinase inhibitor ibrutinib are also promising alternatives. In this summary article, we aim to provide a comprehensive overview of the clinical and molecular background of the disease and potential therapeutic targets, based on the cases reported so far. We diagnosed a patient who, to the best of our knowledge, represents the 748th documented case of this specific pathology. In the context of this patient, we would also like to draw attention to the potential pathogenic role of two novel gene mutations (variants of the MEFV gene “c.2084A>G” and the F2 gene “3′UTR c.*97G>A”).

## 1. Introduction

Schnitzler syndrome (SchS) is an acquired autoinflammatory disorder characterized by persistent urticaria, monoclonal IgM gammopathy, and systemic inflammation. Additional characteristics comprise recurrent fever, arthralgia, and bone pain linked to atypical bone remodeling [1]. While the precise genetic foundation remains ambiguous, certain instances have been associated with the somatic myeloid differentiation primary response 88 (MYD88) L265P mutation, commonly observed in B-cell diseases [2,3,4]. This mutation may indicate a susceptibility to B-cell dysregulation, heightening the risk of lymphoproliferative disorders such as Waldenström’s macroglobulinemia [4]. Notably, although NLRP3 (NOD-, LRR-, and pyrin domain-containing protein 3) mutations are crucial in associated autoinflammatory diseases, they have not been conclusively discovered in SchS.

The genetic basis of SchS is still slightly unclear; however, studies have pinpointed pertinent molecular characteristics. Approximately 30% of patients had the MYD88 L265P mutation, frequently linked to lymphoproliferative diseases [2,3,4]. This mutation may play a role in immunological dysregulation; nevertheless, its existence alone does not entirely elucidate the etiology of the condition.

Unlike other autoinflammatory syndromes, NLRP3 mutations that activate the inflammasome are typically absent in SchS [5]. The clinical response to interleukin (IL)-1 blockers, such as anakinra, indicates that dysregulated inflammasome activation is significant. SchS exhibits similarities to cryopyrin-associated periodic syndromes (CAPS) regarding inflammatory pathways; however, CAPS does not possess the distinctive monoclonal gammopathy present in SchS [5].

Additionally, epigenetic aspects are being investigated. A study found that some female patients had the non-random inactivation of the X chromosome, which suggests that clonal hematopoiesis may be involved [6]. Clonal hematopoiesis, characterized by somatic mutations in hematopoietic stem cells, has been observed in some individuals, suggesting a possible association between age-related immunological dysregulation and the syndrome’s onset, but no definitive epigenetic signature has been established [6,7].

SchS likely embodies a multifaceted interaction of immunological dysregulation, encompassing monoclonal gammopathy, inflammasome activation, and clonal hematopoiesis, and lacking a definitive unifying genetic alteration in all patients. This indicates a combination of acquired immunological modifications rather than a singular monogenic source.

We recently diagnosed a patient with Schnitzler syndrome, which is essentially the focus of this article. Our aim is to provide a brief overview of this distinct type of autoinflammatory disease based on the available, albeit still incomplete, knowledge.

## 2. Epidemiology

Schnitzler syndrome is a rare and underrecognized disease. In 2014, de Koning published a landmark review article summarizing the case reports [8]. However, de Koning’s work did not include new case reports published in 2014 [9,10,11,12,13,14,15,16]. Beyond 2014, numerous case reports were published, some of which dealt with cases diagnosed before 2014. These cases were also, understandably, not included in de Koning’s work. Moreover, some working groups have published some of the case reports and summaries from the period 2014–2024, describing their earlier cases and adding new ones [6,17,18,19,20,21,22,23,24,25,26,27,28,29,30,31,32,33,34,35,36,37,38,39,40,41,42,43,44,45,46,47,48,49,50,51,52,53,54,55,56,57,58,59,60,61,62,63,64,65,66,67,68,69,70,71,72,73,74,75,76,77,78,79,80,81,82,83,84,85,86,87,88,89,90,91,92,93,94,95,96,97,98,99,100,101,102,103,104,105,106,107,108]. After checking the reference lists of available publications and not counting potential overlaps, a total of 747 cases have been published worldwide up to 1 December 2024.

Schnitzler syndrome is present in all ethnicities. The ratio of 1.76:1 indicates a minor predominance of males. The age of patients diagnosed with Schnitzler syndrome varies from 13 to 71 years [109,110]. The average age of onset is approximately 52 years, and it is generally regarded as a late-onset condition. However, the average delay to diagnosis is more than five years [109,110].

## 3. Pathogenesis

### 3.1. Genetic Background

SchS exhibits numerous clinical and biochemical characteristics akin to those of CAPS. Previous studies have associated somatic mutations of NLRP3 with the etiology of SchS and have documented the myeloid lineage mosaicism of NLRP3 in two patients with Schnitzler-like syndrome [111]. These individuals, lacking IgM gammopathy, were probably instances of late-onset mosaic CAPS. Deep next-generation sequencing (NGS) detected no somatic or germline changes in NLRP3 in two extensive datasets [43,86]. In addition, the studies mentioned above did not find any other harmful variants in either NGS panel of genes linked to systemic autoinflammatory illness.

The heterozygous mutation p.(Glu148Gln) of the MEFV (Mediterranean fever) gene has been found in a patient with SchS [105]. This genetic mutation has been linked to familial Mediterranean fever (FMF) in patients exhibiting fever, arthritis, stomach pain, and urticaria [112,113]. The MEFV gene encodes pyrin, a protein that suppresses the inflammasome. There are several documented cases of persistent urticaria associated with mutations in MEFV [114,115,116]. Overactive inflammasomes in FMF lead to higher levels of IL-6, which are associated with changes in the pyrin function. Between crises, these levels return to normal and may be associated with urticaria. However, establishing a causal connection between the heterozygous mutation p.(Glu148Gln) in the MEFV gene and SchS is challenging.

It has been recently demonstrated [117] that MYD88-mediated signaling can activate the promoter of NLRP3, and in the presence of specific NLRP3 promoter sequence variations, it can result in increased NLRP3 promoter activity [118]. The dysregulated expression of NLRP3 may elicit autoinflammatory symptoms. The MYD88-dependent (early-phase) NF-κB (nuclear factor-κB) activity has been shown to increase the transcription of both NLRP3 and IL-1β genes [119]. Moreover, it has been determined that MYD88 deficiency and NF-kB suppression negatively affect the stimulation of NLRP3 protein in response to bacterial products (i.e., lipopolysaccharides (LPSs)). This indicates that signals derived from NF-kB activity regulate NLRP3 expression [120]. Nevertheless, the role of MYD88 and NF-κβ signaling in SchS has not been thoroughly investigated. Over 90% of individuals with Waldenström’s macroglobulinemia exhibit the p.L265P gain-of-function mutation in the MYD88 gene [121]. Some SchS cases have also been reported to display this mutation [59,111]. This mutation activates NF-κB signaling by mimicking the structural consequences of activating phosphorylation [122]. The MYD88 mutation can induce a sustained NF-κB priming signal that enhances the expression of both NLRP3 and IL-1β. Furthermore, the negative-feedback regulation of MYD88 signaling by caspase-1-mediated cleavage has recently been elucidated, and the L265P variation has demonstrated resistance to this caspase-1-mediated suppression [123].

Determining the role of genetic mutations in SchS is not straightforward in several ways. Variants of individual genes often have variable expression and incomplete penetration, which makes it difficult to establish a direct causal link to SchS. Moreover, many autoinflammatory diseases have overlapping clinical features (e.g., fever, rash, arthralgia, elevated IL-1β), making it difficult to identify disease-specific genetic culprits. Most genetic alterations associated with SchS remain largely unexplored.

### 3.2. IL-1 Association

There is no direct evidence for the existence of NLRP3 mutations in SchS. Yet, in SchS, NLRP3 inflammasome activation and the role of IL-1β are characteristic. One of the most obvious pieces of evidence for this is the immediate therapeutic effect of IL-1 inhibitors in the disease. Slightly elevated IL-1β levels have been demonstrated in SchS compared to healthy controls [124]. The interleukin-1 receptor-associated kinase 4 (IRAK-4) is a signal transducer of the Toll-like receptors IL-1R and IL-18R. Monocytes and B cells from patients with SchS have demonstrated the increased phosphorylation of IRAK-4 [125]. The IL-1 inhibitor anakinra treatment reduces phosphorylated IRAK-4 levels to near-normal levels. Despite the elevation of IL-6 levels in SchS [43], the absence of an increase in STAT3 (signal transducer and activator of transcription 3) phosphorylation following anakinra treatment suggests no upregulation of IL-6 signaling.

Elevated IL-18 levels in SchS can also be confirmed compared to healthy controls [43]. While IL-1β is generally barely detectable in plasma, elevated IL-18 levels indicate caspase-1 activation due to the cleavage of its precursor, pro-IL-18 [125]. Elevated levels of extracellular ASCs (apoptosis-associated speck-like proteins containing a CARD) also indicate the NLRP3-mediated activation of pyroptosis [125]. The spontaneous increased production of IL-1α, IL-1β, IL-6, and tumor necrosis factor (TNF)α cytokines by peripheral blood mononuclear cells (PBMCs) in patients with SchS compared to healthy control individuals is indicative of impaired innate immunity [72]. Following LPS stimulation, the levels of these cytokines are even higher [72]. In addition, there is evidence of a difference in the adaptive immune response in patients. After the stimulation of PBMCs with anti-CD3/CD28 beads, the production of interferon (IFN)γ, IL-4, IL-17A, and IL-10 is lower compared to that in healthy controls. This suggests abnormalities in Th1, Th2, Th17, and Treg functioning [72].

**Figure 1:**
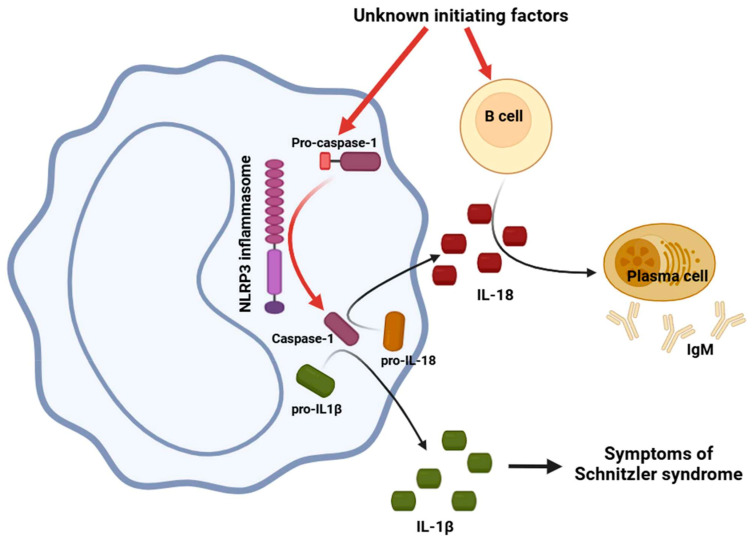
NLRP3 and B cells are activated by an unknown stimulus. The activated NLRP3 cleaves pro-caspase-1 into active caspase-1, which activates inactive IL-1β and IL-18. IL-1β induces cutaneous urticarial rash and other clinical symptoms, while IL-18 promotes the expansion of B-cell clonality and the production of monoclonal antibodies, the majority of which are IgM kappa. NLRP3: NOD-, LRR-, and pyrin domain-containing protein 3; IL: interleukin; IgM: immunoglobulin M. The figure was partially created with https://www.biorender.com (accessed on 2 December 2024).

The etiology of inflammasome function dysregulation in SchS remains unidentified. The condition is marked by the neutrophilic infiltration of the skin, and it is postulated that IL-1β originates from mast cells, similar to CAPS [126,127]. Patients with SchS exhibit skin characterized by neutrophilic leukocytoclasia and NETosis [128,129]. NETosis occurs in the blood of patients, likely exacerbated by cytokines such as IL-1β or IL-6 [128]. The role of NETosis in pathophysiology remains ambiguous, as it can both promote and suppress the inflammatory response [130,131]. CCL2 (C-C Motif Chemokine Ligand 2) is also detectable in the sera of patients with SchS [124]. Following IL-1 stimulation, PMBCs and skin fibroblast cells may synthesize CCL2, which facilitates the recruitment of mononuclear cells to several organs, such as skin and bone tissue. The putative role of interleukins and the NLRP3 inflammasome in SchS is illustrated in Figure 1.

### 3.3. Monoclonal Gammopathy

Monoclonal gammopathy is an essential requirement for diagnosing SchS. IgM (immunoglobulin M) gammopathy is the primary characteristic of the illness, albeit it may sometimes occur alongside IgG and, occasionally, IgA gammopathy [93]. The role of paraprotein remains ambiguous, as it is uncertain whether its emergence is a causative factor or a resultant effect of the disease process.

It is possible to delay the identification of IgM paraprotein for up to four years after the onset of symptoms [25]. Therefore, it is presumed that IgM paraprotein does not function as a pathogenic component in SchS. However, the occasional remission of symptoms during cyclophosphamide +/− rituximab treatment may indicate a pathogenic role for IgM gammopathy [19,132]. The deep sequencing of immunoglobulin heavy chains from individuals with SchS, along with protein microarray analyses of isolated IgM molecules, have not revealed common B-cell clonality [133].

Alternative theories suggest that sustained inflammation leading to plasma cell clonality could be the cause of the emergence of paraproteins. The delayed identification of IgM relative to the symptom start may also indicate this. In SchS, the IL-6 concentrations are heightened. IL-6 serves as a growth and survival factor in plasma cell dyscrasias [134,135]. This might explain why IL-1 inhibitory therapies, which significantly alleviate the symptoms of SchS, exert no substantial impact on the monoclonal component and fail to avert the onset of lymphoproliferative illness.

Some theories propose that the development of paraprotein parallels the pathophysiology associated with inflammasome dysfunction. The aforementioned MYD88 L265P mutation may represent a shared characteristic between the two disorders. The MYD88 gene mutation in IgM monoclonal gammopathy of undetermined significance (MGUS) correlates with a 20-fold heightened chance of developing Waldenström’s macroglobulinemia [136]. Nonetheless, as this mutation is not routinely observed in SchS, numerous other unidentified variables may be linked to the heightened activation of the NF-κB pathway.

**Figure 2:**
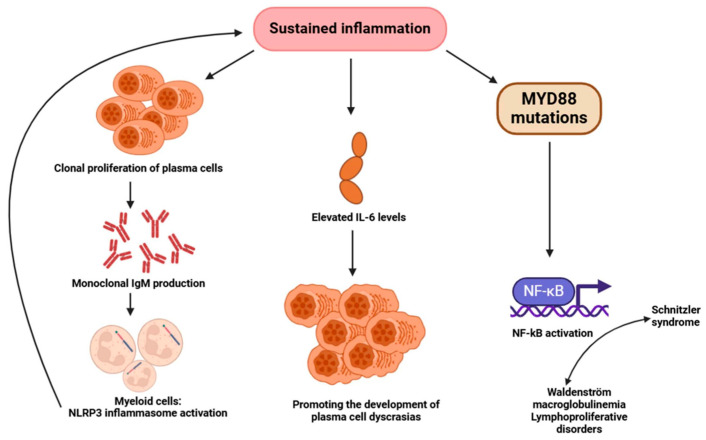
Proposed correlations between sustained inflammation and monoclonal IgM production. Chronic inflammation promotes the clonal expansion of plasma cells. This could result in the synthesis of monoclonal IgM, leading to the activation of the NLRP3 inflammasome in myeloid cells. This exacerbates chronic inflammation. Chronic inflammation elevates IL-6 concentrations. This may consequently promote the emergence of plasma cell dyscrasias. Through activating NF-kB, the MYD88 mutation makes a link between Schnitzler syndrome, Waldenström disease, and other lymphoproliferative diseases. NLRP3: NOD-, LRR-, and pyrin domain-containing protein 3; IL: interleukin; IgM: immunoglobulin M; MYD88: myeloid differentiation primary response 88; NF-kB: nuclear factor-kB. The figure was partially created with https://www.biorender.com (accessed on 2 December 2024).

Finally, observations in Waldenström’s macroglobulinemia suggest that monoclonal IgM, the principal diagnostic element of SchS, may identify autoantigens on myeloid cells, thereby initiating NLRP3 inflammasome activation linked to the production of proinflammatory cytokines, particularly interleukin-1β [137]. Therefore, one cannot rule out the possibility that lowering monoclonal protein could still lead to a favorable clinical outcome (Figure 2).

## 4. Clinical Aspects

### 4.1. Disease Manifestations

A majority of patients exhibit recurrent fever. Body temperature may exceed 40 °C; however, it is typically easily tolerated without accompanying chills. Fever episodes may be associated with a rash or musculoskeletal discomfort. The frequency of fever episodes varies, ranging from daily to a few times a year, without following a consistent pattern [8].

A recurrent urticarial rash is often the initial sign, perhaps occurring months or even years before additional symptoms manifest. This unusual rash consists of pink-to-red, slightly elevated papules or plaques with no alteration in the skin surface. The lesions are uniform, confluent, and situated over the limbs and trunk, frequently excluding the face, palms, and soles. Individual lesions persist for less than 24 h and heal without leaving scars. One may observe a halo of vasoconstriction and dermographism, but angioedema rarely occurs. Unlike classic urticaria, pruritus is typically absent. A subgroup of people may experience mild itching or burning over time. Antihistamines are ineffectual. The frequency of eruptions is inconsistent, varying from daily occurrences to several instances annually [1,8].

Histopathological analysis indicates a neutrophilic urticarial dermatosis [138]. Neutrophils infiltrate the dermis surrounding blood vessels and inside the interstitial space, accompanied by leukocytoclasis. Minimal-to-absent edema and no evidence of vasculitis are present. One should not confuse the perivascular neutrophilic infiltrate associated with leukocytoclasis with vasculitis, as neutrophilic urticarial dermatosis does not exhibit fibrinoid alterations in the vessel walls. Particularly indicative is neutrophilic epitheliotropism, especially in the vicinity of the sweat glands. SchS is not the only condition that exhibits the histopathological pattern of neutrophilic urticarial dermatosis; it can also manifest in CAPS, adult-onset Still’s disease, or systemic lupus erythematosus [138].

Musculoskeletal involvement is another characteristic that impacts over two-thirds of patients. Non-erosive arthritis is an exceptional condition that should raise questions about the diagnosis, despite the fact that joint pain is commonly experienced. The lower extremities (tibia, femur, and pelvis) are the most common sites of bone pain, although it may also manifest in the spine, forearm, or clavicle. Prior to the manifestation of clinical symptoms, radiographic abnormalities have been documented. The sclerotic lesions and other imaging abnormalities are not specific, as they may be observed in other dysplastic or infiltrative diseases, such as sclerotic myeloma, Erdheim–Chester disease (ECD), systemic mastocytosis, endochondroma, or POEMS (polyneuropathy, organomegaly, endocrinopathy, M-protein, skin changes) [11]. The most lesions exhibit both sclerosis and lysis. Conventional radiology is less sensitive than bone scintigraphy in identifying lesions [139]. The femur and tibia are the most frequently encountered bones, along with the humerus, radius, ulna, fibula, and pelvic bones. The “hot knee” symptom, which is equivocal for both SchS and ECD, is characterized by the involvement of the distal femur and proximal tibia [11]. The administration of anti-IL-1 to patients led to a significant reduction in bone scan abnormalities, with some patients experiencing complete resolution [138]. MRI can also detect sclerosis, which can range from a mild diffuse medullary signal to extensive medullary edema [27]. Fluorodeoxyglucose (FDG)-positron emission tomography (PET) can also be used to visualize lesions, demonstrating an increase in FDG absorption at sites of bone sclerosis. Pathologic examinations are frequently unremarkable or may demonstrate nonspecific inflammation or sclerosis [8].

Approximately one-quarter of patients exhibit enlarged lymph nodes, while hepatomegaly and splenomegaly occur in a limited subset of patients [8]. Lymph node enlargement is typically located in the axillary or groin regions. They may be numerous, persistent, and indicative of a lymphoproliferative disease. The lymph node biopsy indicates reactive lymphadenitis.

Fatigue, loss of weight, and myalgia are prevalent clinical manifestations. A minority of patients have documented neuropathy, primarily symmetrical sensory polyneuropathy. There is a documented case of Schnitzler syndrome and aortitis, both of which respond quickly to IL-1 blocking [40].

### 4.2. Laboratory Findings

The existence of a monoclonal gammopathy is an essential criterion for SchS. A total of 85% of patients with SchS have monoclonal IgM gammopathy with a kappa light chain. Only a limited number of cases, either in isolation or in conjunction with an IgM component, have reported monoclonal IgG gammopathy [93,140].

Inflammatory indicators, including C-reactive protein (CRP) and the erythrocyte sedimentation rate (ESR), are typically high. Neutrophil and white blood cell overproduction is often seen on a full blood count. However, inflammatory anemia and thrombocytosis can happen when there is long-term inflammation. Elevated alkaline phosphatase levels may result from improper bone remodeling. Complement levels are either normal or high, unlike hypocomplementemic urticarial vasculitis or cryoglobulinemic vasculitis [1,8].

A crucial aspect distinguishing SchS from autoimmune disorders is the absence of autoantibodies often associated with autoimmune conditions, such as antinuclear antibodies (ANAs), rheumatoid factor (RF), or other specific autoantibodies, in the sera of affected patients. The immunological anomalies associated with SchS predominantly involve monoclonal gammopathy, primarily IgM and, to a lesser extent, IgG. The occurrence of autoantibodies, such as ANA positivity, during the progression of the disease is extremely rare. If it does occur, it is likely due to a secondary anomaly or coincidence [141], and it typically results from the autoantibody activity of the monoclonal component [142].

Regarding bone formation, patients with SchS display high blood levels of bone-specific alkaline phosphatase and osteocalcin. In contrast, low levels of the C-terminal telopeptide of type 1 collagen and the soluble ligand of the receptor activator of nuclear factor-B indicate reduced bone resorption [143].

### 4.3. Complications

The primary consequence of SchS is the onset of lymphoproliferative disorders, predominantly Waldenström’s macroglobulinemia. The precise incidence of progression is unknown due to the rarity of the condition. In a median follow-up period of eight years, hematological malignancy occurred in 35 of 281 patients [8]. Nonetheless, the actual incidence is likely greater. Most studies have demonstrated that IL-1 and IL-6 inhibitors neither alter monoclonal gammopathy nor prevent the development of lymphoproliferative diseases. The extended monitoring of monoclonal gammopathy in individuals with SchS, therefore, is essential.

The chronic inflammation-induced overproduction of serum AA protein can lead to the infrequent complication of AA amyloidosis, particularly in patients with undiagnosed and therefore untreated SchS [8,144,145]. However, studies have demonstrated the effectiveness of IL-1 inhibitor treatment in preventing AA amyloidosis.

To date, there has been only one case worldwide of the spontaneous sustained remission of SchS [146]. This patient was initially administered a mixture of two antihistamines, yielding no substantial improvement. The administration of 5 mg of prednisone resulted in partial symptom amelioration. Prednisone was gradually reduced and subsequently discontinued, resulting in the continuation of arthralgias, bone discomfort, and intermittent urticaria. Subsequently, the patient was administered intermittent aspirin or nonsteroidal anti-inflammatory medicines. Four years post-disease onset, remission of the illness, alongside the resolution of the monoclonal gammopathy, was observed.

## 5. Diagnostic Criteria

Lipsker et al. established the original diagnostic criteria, which were subsequently amended by de Koning et al. [110,147] (Table 1). A diagnosis of SchS necessitates the fulfillment of both primary criteria and a minimum of two secondary criteria, subsequent to the exclusion of alternative diagnoses. The primary requirements are urticarial skin rash and the monoclonal IgM component (or IgG: variant type). The minor criteria are intermittent fever, arthralgia or arthritis, bone pain, lymphadenopathy, hepato- and/or splenomegaly, increased ESR and/or leukocytosis, and bone abnormalities identified through radiographic or histological examination.

The Strasbourg criteria (Table 1), derived from the Lipsker criteria, establish two mandatory features: “chronic urticarial rash” and “monoclonal IgM or IgG” [148]. For a definitive diagnosis of SchS, two mandatory criteria must be met, along with a minimum of two minor requirements if the monoclonal is IgM. If the monoclonal is IgG, it must meet three minor criteria. For a probable diagnosis of SchS, two mandatory criteria must be met, along with at least one minor criterion for the IgM monoclonal or two minor criteria for the IgG monoclonal. Major criteria consist of a persistent urticarial rash and monoclonal IgM or IgG. Minor criteria include recurring fever, objective indications of aberrant bone remodeling with or without bone pain, a neutrophilic dermal infiltration on a skin biopsy, or leukocytosis and/or increased CRP.

In 2017, both of the diagnostic criterion systems were subsequently validated in a group of 42 individuals with pre-existing diagnoses [34]. The reliability of these criteria has not been assessed in cases of recent-onset illness. Patients may not completely satisfy all criteria at presentation. In instances of neutrophilic urticarial dermatosis linked to IgM monoclonal gammopathy, SchS should be considered, regardless of the inadequacy of the minor criteria.

### Differential Diagnosis

The differential diagnosis of SchS is crucial, as symptoms may also manifest across multiple other conditions [1,8]. Table 2 encapsulates the principal syndromes and their defining traits.

## 6. Therapeutic Possibilities

Before the introduction of IL-1 inhibitors, SchS was a challenging condition to manage. Various therapies have been employed, although none have succeeded in achieving a sustained remission, except for excessively large and extended steroid dosages [8,149]. Therapeutic trials demonstrating modest efficacy have been conducted with the following agents: rituximab, IFNα, thalidomide, colchicine, pefloxacin, cyclosporine, and PUV-A1 (psoralen and ultraviolet A1). Therapeutic interventions utilizing the following agents have proven to be either entirely ineffective or minimally effective: azathioprine, TNFα inhibitors, chloroquine, sulfasalazine, fludarabine, UV-A or UV-B phototherapy, sulphones, leflunomide, alkylating agents, cyclooxygenase (COX) inhibitors, hydroxychloroquine, dapsone, histone deacetylase inhibitor (ITF2357), doxepin, bisphosphonates, intravenous immunoglobulins, psoralen, H1 antihistamines, plasmapheresis, extracorporeal immunoadsorption, bortezomib, and dihydroergotamine [8,150].

IL-1 inhibitors have significantly transformed the treatment of these individuals. They are now the primary treatments for SchS, demonstrating significant efficacy, a quick response, and few adverse effects. In addition to anti-IL-1Ra (anakinra), anti-IL-1β antibodies (canakinumab), the fusion protein IL-1R (rilonacept), anti-IL-6 antibodies (tocilizumab), and an irreversible inhibitor of Bruton’s tyrosine kinase (BTK) (ibrutinib) have also demonstrated efficacy [8,150].

The effectiveness of anakinra in SchS was initially documented in 2005 [149]. Since that time, it has emerged as the primary treatment for the condition. Anakinra administered subcutaneously has a half-life ranging from 4 to 6 h. Even though symptoms resolve within a few hours post-injection, failure to sustain treatment leads to recurrence within 48 h. Consequently, daily (sometimes bi-daily) injections are necessary to sustain remission. Canakinumab has a significantly extended half-life, spanning from 22.9 to 26 days. A randomized, placebo-controlled trial established the efficacy and safety of canakinumab in SchS [45]. Additionally, a four-year extension study with a 62-day interval between injections validated a sustained impact [151]. In comparison to anakinra, canakinumab has the distinct benefit of a reduced dose frequency. Anakinra, due to its brief half-life, is preferred in the event of potential complications. Despite anakinra’s cheaper price compared to that of canakinumab, the total treatment expense may be significantly greater due to its frequent administration.

A single-center, open-label research study with eight participants demonstrated the effectiveness of rilonacept [152]. The study administered a solitary dose of 320 mg to induce remission and continued to administer 160 mg of rilonacept weekly for 12 months to maintain remission. The rilonacept treatment led to a swift clinical response, a persistent decrease in inflammatory markers, and no major adverse events associated with it.

An open-label research study with nine participants assessed the effectiveness of tocilizumab [153]. The majority of patients exhibited a clinical and biochemical response after the weekly subcutaneous administration of 162 mg of tocilizumab. A decline in the treatment efficacy was noted over time. Conversely, case studies indicate that four individuals with SchS attained complete remission with tocilizumab [65,154].

Ibrutinib is a non-reversible inhibitor of BTK licensed for treating various lymphoproliferative diseases. BTK has been identified as a regulator of the NLRP3 inflammasome, and in vitro inhibition by ibrutinib has demonstrated a reduction in the production of IL-1β from immune cells [154]. Several case reports have documented the partial or complete effectiveness of ibrutinib [50,77,85,155,156].

## 7. Our Own Experience with Schnitzler Syndrome

**Figure 3:**
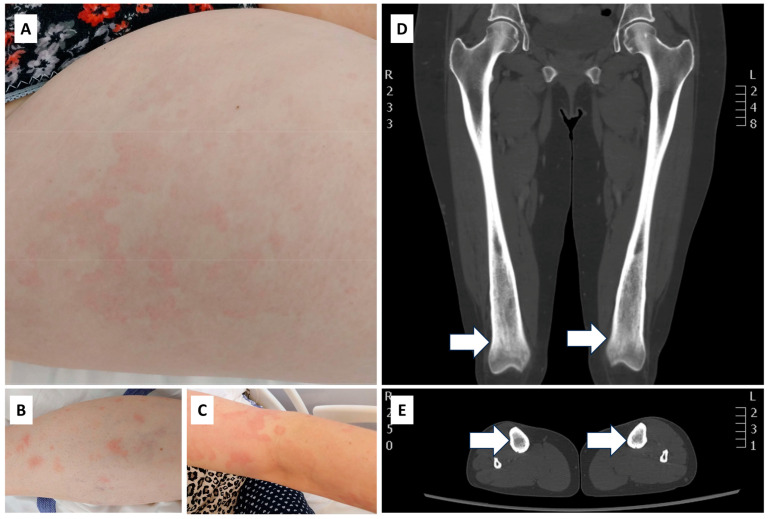
The patient with Schnitzler syndrome has urticarial rashes on her thigh (**A**), leg (**B**), and forearm (**C**). Coronal CT scan of the thighs (**D**) indicated ill-delineated marrow infiltration (arrows) in the distal metaphyses and epiphyses of both femurs and vague intramedullary sclerosis (arrows) of the proximal tibias (**E**). L: left, R: right, CT: computed tomography.

The basis for writing this review article is that we also confirmed SchS in one of our female patients this year. The 54-year-old female patient’s complaints/symptoms started in 2020 as chronic, recurrent, non-itching, and sometimes giant urticariform rashes (Figure 3A–C); periodic high fevers; night sweating; diffuse arthralgia; and, in the last period, progressive bone pains affecting both sides of the tibia. Several institutions have examined her in recent years, finding no abnormalities suggestive of infection, malignancy, or definitive autoimmune disease. The total IgE level was normal, and an epicutaneous patch test was negative. A gynecological examination revealed a myomatous uterus.

Our clinic’s Immunology Department first detected the patient in April 2024. The patient’s detailed findings highlighted the following abnormalities: an accelerated erythrocyte sedimentation rate (ESR: 101 mm/h), elevated CRP (64 mg/L), moderate microcytic anemia with leukocytosis and thrombocytosis (WBC: 12.14 G/L; RBC: 4.24 T/L; hgb: 103 g/L; HTC: 0.34 L/L; MCV: 79 fL; PLT: 445 G/L), and monoclonal IgM proliferation (14.5 g/L, IgM kappa type). Extended immunoserology [i.e., ANA panel, anti-dsDNA, anti-extractable nuclear antigen (ENA) panel, anti-cardiolipin (CL), anti-b2GPI (beta2 glycoprotein 1), anti-tTG (anti-tissue transglutaminase antibody), ACPA (anti-citrullinated peptide antibody), ANCA (antineutrophil cytoplasmic antibodies), and organ-specific autoantibodies] was negative. Her illness was evaluated as periodic fever syndrome with an autoinflammatory background.

We admitted her to the Immunology Department for further investigation between 15 and 21 May 2024. A bone marrow trephine biopsy and aspirate samples upon histology, immunohistochemistry, and cytology indicated no signs of any lymphoproliferative abnormalities or hematological malignancies. The whole-body computed tomography (CT) scan revealed predominantly sclerotic bony lesions, indicating abnormal bone remodeling, in both the patient’s distal femurs and proximal tibias (Figure 3D,E). The scan also revealed axillary, periportal, paraaortic, parailical, and inguinal lymphadenopathy, along with a mild degree of splenomegaly. A skin biopsy suggested urticaria without any signs of vasculitis. A direct immunofluorescence examination (DIF) revealed nonspecific C3 and IgM granular precipitation along the basement membrane. The serum IL-6 level was elevated (19.5 ng/L). We also performed detailed postinfection serological tests [including human immunodeficiency virus (HIV)-1 and -2; Treponema pallidum; hepatitis B surface antigen (HBsAg); hepatitis B core antigen (HBcAg); anti-HCV (hepatitis C virus); anti-HEV (hepatitis E virus)] and a sexually transmitted disease (STD) polymerase chain reaction (PCR) panel (including Neisseria gonorrhoea, Mycoplasma genitalium, Chlamydia trachomatis, and Ureaplasma sp.), which yielded negative results. Due to bone pain, we started symptomatic tramadol administration, but after it proved ineffective, we successfully switched to a meloxicam and metamizole combination with PPI protection. We also conducted a hemoculture during her fever, which yielded negative results.

In conclusion, we excluded the presence of a “classic” systemic autoimmune disease in this patient. However, the patient was clearly diagnosed with SchS according to both the Lipsker and Strasbourg criterion systems. We confirmed that SchS was responsible for the clinical picture, which included recurrent urticariform exanthemas, recurrent fevers, diffuse arthralgia, osseous pain, osteosclerosis, and extensive lymphadenopathy.

Based on the results of the genetic testing performed, no clinically relevant findings were identified in the NLRP3 and MYD88 genes.

In contrast, the autoinflammatory disease-related gene panel (Supplementary Table S1) identified two pathogenic/probably pathogenic mismatches in the MEFV gene c.2084A>G (also known as p.Lys695Arg or rs104895094), while in the prothrombin F2 gene, the 3′UTR c.*97G>A variant (also known as rs1799963) was detected.

Two variants of uncertain significance were also identified, which showed weak associations with the inheritance, phenotype, and variant classification. These were ITGB2 c.1570 G>A (also known as p.Val524Ile or rs201654243) and C2: c.841_868del (also known as p.Val281Profs*110 or rs9332736).

Remission induction treatment was conducted using the fully human monoclonal antibody anti-IL-1β, canakinumab. We provided two subcutaneous injections of 150 mg of canakinumab seven days apart without severe problems. After three days of the initial medication dose, the patient’s complaints and symptoms subsided, her cutaneous symptoms disappeared, she became afebrile, her bone pain was significantly reduced, and her inflammatory markers decreased. Subsequent to the second dose, she experienced no further symptoms. The patient is in complete clinical remission on maintenance therapy with 150 mg of canakinumab administered every 8 weeks. Monoclonal IgM levels stabilized at around 11 g/L after a temporary spike (15.4 g/L) post-induction therapy.

To the best of our knowledge, the case we have described is the 748th documented case of Schnitzler syndrome worldwide.

## 8. Discussion

Schnitzler syndrome is an unusual, late-onset acquired autoinflammatory disorder. Patients with a resistant chronic urticarial rash, persistently elevated inflammatory parameters, bone pain, and recurrent episodes of fever associated with IgM monoclonal gammopathy should consider Schnitzler syndrome. SchS’s rash differs from typical urticaria due to its lack of edema, absence of itching, and neutrophilic histological characteristics. While the treatment with IL-1 inhibitors has significantly enhanced the quality of life for patients, it does not impact the monoclonal component. More is being learned about the physiopathology of Schnitzler syndrome, but the main question about how its autoinflammatory features are connected to monoclonal gammopathy is still open.

The exact role of the genetic differences we have identified in SchS is not yet known. In the MEFV gene, the c.2084A>G variant results in the amino acid substitution p.Lys695Arg (also known as K695R). The p.Lys695Arg (K695R) variant is located in the B-box domain of pyrin, which is key in the regulation of inflammasome activity [157,158]. Particularly in the Mediterranean region, the K695R variant is relatively common and frequently detected in mild to atypical forms of FMF [159]. Despite being previously considered pathogenic, the literature represents K695R as a variant of unknown significance (VUS), usually not sufficient on its own to form the classical FMF clinical picture.

The prothrombin gene (F2), and specifically its 3′UTR c.*97G>A variant (rs1799963, commonly referred to as G20210A), is widely recognized for its correlation with heightened thrombotic events [160,161]. While inflammatory diseases are not directly associated with this variant, it is crucial to take into account the following factors in the context of SchS. The G20210A variant is located in the 3′ non-coding region of the prothrombin gene and results in increased prothrombin levels in plasma [161]. This can lead to hypercoagulability and an increased risk of thrombosis. This variant is one of the most common genetic causes of congenital thrombophilia, especially in the European population. Inflammatory activity is common in SchS and may itself result in a prothrombotic state (e.g., due to increased inflammatory cytokines such as IL-1 and IL-6). If a patient carries the G20210A variant, this may further increase the risk of thrombosis, especially during systemic inflammatory episodes. Despite its primary impact on the coagulation cascade, G20210A could potentially influence the inflammatory response in SchS, given the strong correlation between coagulation and inflammation. Fibrinogen, thrombin, and other coagulation factors can also serve as inflammatory mediators, potentially influencing the inflammasome.

The diagnosis of SchS is currently based on clear clinical and laboratory criteria, so genetic testing is not necessary in routine diagnostic practice. The targeted inhibition of IL-1β overproduction (e.g., by IL-1 receptor antagonists or anti-IL-1β monoclonal antibodies), which is central to the pathomechanism of the disease, is independent of underlying genetic abnormalities and is currently the basis of treatment.

However, there are several reasons why the wider application of genetic testing could be important. The identification of new genetic variants may help us understand the molecular background of the disease and identify new therapeutic targets. Furthermore, the possibility of genetic- or epigenetic-based interventions could open up new avenues for disease treatment in the longer term. Finally, the persistence of monoclonal gammopathy in the presence of anti-IL-1 treatments underscores the risk of the malignant transformation toward lymphoma, highlighting the need for the further exploration of its genetic basis.

Given the infrequent occurrence of SchS, it would be beneficial to identify biomarkers that could speed up diagnosis and predict treatment outcomes. Consequently, additional investigation into the influence of genetic variations and epigenetic differences on the inflammasome pathway is necessary. Organizing clinical studies to evaluate novel anti-inflammatory medicines, such as IL-18 and IL-33 inhibitors, may potentially be beneficial. Nonetheless, considering the restricted experience with the condition, it is equally vital to emphasize the advancement of telemedicine. In rare diseases, the accessibility of remote consultations and specialist networks helps expedite diagnosis.

## Figures and Tables

**Table 1:** The diagnostic criterion systems of Schnitzler syndrome.

Diagnostic Criterion Systems of Schnitzler Syndrome		
	Lipsker	Strasbourg
**Major (obligate) criteria**	Urticarial rash	Chronic urticarial rash
	Monoclonal IgM component	Monoclonal IgM or IgG component
**Minor criteria**	Fever	Recurrent fever (> 38°C, otherwise unexplained)
	Arthralgia or arthritis	Objective findings of abnormal bone remodeling with or without bone pain **
	Bone pain	Neutrophilic urticarial dermatosis on skin biopsy
	Palpable lymph nodes	Neutrophils > 10,000/mm^3^ and/or CRP > 30 mg/L
	Liver or spleen enlargement	
	Elevated ESR	
	Leukocytosis	
	Abnormal findings on bone morphologic investigations	
**Definite diagnosis**	Two major criteria AND at least two minor criteria *	Two obligate criteria AND at least two minor criteria if IgM and three minor criteria if IgG
**Probable diagnosis**		Two obligate criteria AND at least one minor criterion if IgM and two minor criteria if IgG

* A swift and quick reaction in patients administered IL-1 inhibitors corroborates the diagnosis. Should the patient not respond to anakinra, a reevaluation of the diagnosis is required. ** Assessed by bone scintigraphy, MRI, or elevation of bone alkaline phosphatase.

**Table 2:** The differential diagnosis for Schnitzler syndrome.

Differential Diagnosis of Schnitzler Syndrome			
Disease Groups	Disorders		Characteristics
**Autoinflammatory diseases**	Cryopyrin-associated periodic syndromes (CAPS) including familial cold urticaria, Muckle–Wells syndrome, CINCA (chronic infantile neurological cutaneous and articular syndrome), and neonatal-onset multisystem inflammatory disease (NOMID)		Cold-induced symptoms, neurological involvement (e.g., headache, meningitis), often starting at a young age. Negative monoclonal gammopathy but elevated inflammatory markers.
**Hematological diseases**	Waldenström macroglobulinemia		Monoclonal IgM is present, but no urticarial rash or autoinflammatory component. Often associated with lymph node, spleen enlargement, and B symptoms.
	Monoclonal gammopathy of unknown significance (MGUS)		Monoclonal IgM may be present but without inflammatory symptoms and urticaria.
**Autoimmune diseases**	SLE (systemic lupus erythematosus)		Urticarial rash may be present, but autoantibodies such as ANA or double-stranded (ds)DNA positivity are characteristic. Multisystemic involvement, such as renal system or arthritis, which is not characteristic of Schnitzler syndrome.
	Hypocomplementemic urticarial vasculitis syndrome (HUVS)		Similar to urticaria but characterized by signs of complement deficiency and vasculitis. Often associated with angioedema and other organ involvement (e.g., kidney).
**Diseases of infectious origin**	Cryoglobulinemia (associated with hepatitis C)		Similar skin lesions but may have the presence of cryoglobulins and signs of liver damage.
	Endocarditis or osteomyelitis		Long-standing, low-grade inflammation, but these are more likely to have an infectious source.
**Paraneoplastic syndromes**			Cutaneous paraneoplastic syndromes, which may rarely mimic urticaria-type skin symptoms.
**Other pathologies**	Chronic idiopathic urticaria		Similar skin lesions but lacking monoclonal gammopathy and elevation of inflammatory markers.
	Still’s disease in adulthood		Similar inflammatory signs, but typically elevated ferritin levels and a different clinical presentation (e.g., characteristic febrile episode).

## Data Availability

The original contributions presented in this study are included in the article/Supplementary Material. Further inquiries can be directed to the corresponding author(s).

## Supplementary Materials

The following supporting information can be downloaded at https://www.mdpi.com/article/10.3390/ijms26020598/s1.
