# Supplementary material for: Background and Clinical Features of a Unique and Mysterious Autoinflammatory Disease, Schnitzler Syndrome
Source: Int J Mol Sci. 2025 Jan 12;26(2):598. doi: 10.3390/ijms26020598 (PMC11765222; doi:10.3390/ijms26020598)
Supplement: Supplementary file 1 [file ijms-26-00598-s001.zip › ijms-3384004-supplementary.pdf]

Supplementary Table S1.

The list of 195 autoinflammatory disease-related genes that were tested with full EXOM analysis by Illumina new-generation sequencing is as follows
ACP5, ADA, ADA2, ADAM17, ADAR, AICDA, AIRE, ALPI, ALPK1, ANKZF1, AP1S3, AP3B1, AP3D1, ARPC1B, ASAH1, BACH2, BLOC1S6, BTK, C1QA, C1QB, C1QC, C1R, C1S, C2, CARD11, CARD14, CARD8, CASP10, CASP8, CCBE1, CD27, CD3G, CD40, CD40LG, CD48, CD55, CDC42, CEBPE, COPA, CR2, CTLA4, CYBA, CYBB, CYBC1, DCLRE1C, DDX58, DEF6, DKC1, DNASE1, DNASE1L3, DNASE2, DOCK8, DSG1, DUOX2, ELANE, ELF4, F2, FADD, FAS, FASLG, FCHO1, FOXP3, G6PC3, GATA2, HAVCR2, ICOS, IFIH1, IKBKG, IL10, IL10RA, IL10RB, IL1RN, IL21, IL21R, IL2RA, IL2RB, IL2RG, IL36RN, IRF2BP2, ISG15, ITCH, ITGAM, ITGB2, ITK, JAK1, LACC1, LIG4, LPIN2, LRBA, LSM11, LYN, LYST, MAGT1, MEFV, MVK, NCF2, NCF4, NCSTN, NFAT5, NFKB1, NFKB2, NFKBIA, NLRC4, NLRP1, NLRP12, NLRP3, NLRP7, NOD2, OAS1, ORAI1, OTULIN, PEPD, PIK3CD, PIK3R1, PLCG2, PNP, POLA1, POMP, PRF1, PRG4, PRKCD, PSENEN, PSMA3, PSMB10, PSMB4, PSMB8, PSMB9, PSMG2, PSTPIP1, RAB27A, RAC2, RAG1, RAG2, RASGRP1, RBCK1, RELA, RFX5, RFXANK, RFXAP, RIPK1, RMRP, RNASEH2A, RNASEH2B, RNASEH2C, RNF31, RNU7-1, RTEL1, SAMD9L, SAMHD1, SCO2, SH2D1A, SH3BP2, SI, SIAE, SKIV2L, SLC29A3, SLC37A4, SLC7A7, STAT1, STAT2, STAT3, STAT4, STAT5B, STIM1, STX11, STXBP2, TBX1, TGFB1, TGFBR1, TGFBR2, TLR7, TMEM173, TNFAIP3, TNFRSF11A, TNFRSF13B, TNFRSF13C, TNFRSF1A, TNFRSF6B, TNFSF12, TOP2B, TPP2, TRAP1, TREX1, TRNT1, TTC37, TTC7A, UBA1, UNC13D, UNG, USP18, WAS, WDR1, XIAP, ZAP70, ZNF341
